# ETS Exposure and PAH Body Burden in Nonsmoking Italian Adults

**DOI:** 10.3390/ijerph15061156

**Published:** 2018-06-01

**Authors:** Laura Campo, Elisa Polledri, Petra Bechtold, Giulia Gatti, Giulia Quattrini, Luca Olgiati, Michael Romolo, Andrea Ranzi, Paolo Lauriola, Giuliano Carrozzi, Silvia Fustinoni

**Affiliations:** 1 Department of Clinical Sciences and Community Health, University of Milan and Fondazione IRCCS Ca’ Granda Ospedale Maggiore Policlinico, 20122 Milano, Italy; laura.campo@policlinico.mi.it (L.C.); elisa.polledri@policlinico.mi.it (E.P.); luca.olgiati@policlinico.mi.it (L.O.); 2 Department of Public Health, Local Health Unit, 41121 Modena, Italy; p.bechtold@ausl.mo.it (P.B.); gi.gatti@ausl.mo.it (G.G.); g.quattrini@ausl.mo.it (G.Q.); michael.romolo@regione.emilia-romagna.it (M.R.); g.carrozzi@ausl.mo.it (G.C.); 3 Environmental Health Reference Centre, Regional Agency for Environmental Prevention of Emilia Romagna, 41121 Modena, Italy; aranzi@arpae.it; 4 Italian National Research Council, Institute of Clinical Physiology, Unit of Environmental Epidemiology and Disease Registries, 56124 Pisa, Italy; paolo.lauriola@gmail.com

**Keywords:** environmental tobacco smoke, urinary cotinine, polycyclic aromatic hydrocarbons, human biomonitoring, 1-hydroxypyrene

## Abstract

Active smoking is associated with increased body burden of polycyclic aromatic hydrocarbons (PAHs); the aim of this study was to assess whether environmental tobacco smoking (ETS) increases the internal dose of PAHs. In 344 nonsmoking Italian adults, out of 497 individuals selected as representative of the population of the town of Modena, ETS exposure was evaluated by a self-administered questionnaire and by the measurement of urinary cotinine (COT-U). PAH exposure was assessed by the measurement of urinary 1-hydroxypyrene (1-OHPYR) and of ten urinary PAHs. In all subjects, median (5th–95th percentile) COT-U was 0.47 (<0.1–3.91) µg/L. While 58 subjects reported to be ETS exposed (ETS_QUEST_), 38 individuals were identified as ETS exposed on the basis of a COT-U value of 1.78 (90% confidence interval 1.75–1.80) µg/L, previously derived as an upper reference value in not ETS exposed Italian adults (ETS_COT_). Median COT-U levels were 1.38 (<0.1–9.06) and 3.63 (1.80–17.39) µg/L in ETS_QUEST_ and in ETS_COT_ subjects, respectively. Significant correlations between COT-U and 1-OHPYR, and urinary anthracene, fluoranthene, pyrene, and chrysene were found among all subjects. Significantly higher levels of 1-OHPYR, and urinary fluorene, anthracene, and pyrene were found in ETS_COT_ individuals. The results of multiple linear regression analyses, taking into consideration diet and other sources of PAHs exposures such as the residence area/characteristics and traffic, confirmed that 1-OHPYR and urinary fluorene were affected by ETS exposure, even if ETS played a minor role.

## 1. Introduction

Environmental tobacco smoke (ETS), also called passive smoke or involuntary smoking, is made of several components: secondhand smoke, that is the combination of the mainstream smoke exhaled by the smoker and of side-stream smoke released by the burning of the tobacco product, and thirdhand smoke, that is residual tobacco smoke pollutants remaining on surfaces and dust after tobacco has been smoked and that react with oxidants and other compounds in the environment to form secondary pollutants [1].

More than 4000 chemicals have been identified in ETS. Moreover, time and environmental conditions make ETS composition and properties different from that of mainstream and side-stream smoke. In particular, physicochemical properties of ETS vary in terms of both the proportion of constituents in the particulate or in the vapor phase of smoke and the particle size. Moreover, the concentrations of the individual constituents are different due to their dilution in the indoor environment and to secondary reactions [2]. ETS, as well as active smoking, has been classified as carcinogenic to humans, based on sufficient evidences of causing lung cancer, by the International Agency for Research on Cancer (IARC) [2].

Among those chemicals contributing to ETS composition are Polycyclic Aromatic Hydrocarbons (PAHs), which are a complex class of more than one hundred compounds formed by the incomplete combustion of organic materials. PAHs, which have been identified in mainstream and in higher amounts in side-stream smoke [3,4], have various chemical and toxicological properties. Some PAHs have been classified as human carcinogens [5]; moreover, PAH exposure is associated with negative reproductive, neurodevelopmental, cardiovascular, and inflammatory effects [6,7,8,9].

While the contribution of active tobacco smoke to PAH body burden is well known [10], few data are available for ETS exposure [11,12]. Possible reasons may be found in the difficulty encountered in defining ETS exposure as several variables, among which the ETS source, duration of exposure, distance from the source, presence and effectiveness of a ventilation system, and personal characteristics, affect the assessment of the inhaled dose. Moreover, as PAHs are ubiquitous pollutants of living and occupational environments, possible sources of co-exposure should be taken into account in assessing personal PAH exposure, such as the residence zone, traffic exposure, diet, cooking habits, and possible occupational exposure.

PAH exposure can be assessed by human biomonitoring (HBM). PAHs, once absorbed in the body are readily metabolized to phenols that are excreted in urine and feces as glucuronyl or glutathione conjugates [13,14]. A small percentage of absorbed PAHs is excreted unmetabolized [15,16]. A very interesting approach in the biological monitoring of PAH exposure is given by the obtainment of a global profile of several urinary compounds that would be useful for obtaining a better characterization of the exposure.

The aim of this study was to assess whether ETS increases the internal dose of PAHs in a representative population of adults, using data that had been collected previously to study the exposure to emissions from a local urban-waste incineration plant [17]. ETS exposure was evaluated by both self-reporting and measurement of urinary cotinine (COT-U), a sensitive marker of both active and passive smoke, for which we had previously derived an upper reference value of 1.78 µg/L, in not ETS exposed Italian adults [18]. To evaluate PAH exposure, the measurement of urinary 1-hydroxypyrene (1-OHPYR), a routinely used biomarker of PAHs exposure, and of ten unmetabolized PAHs (naphthalene, acenaphthylene, acenaphthene, fluorene, phenanthrene, anthracene, fluoranthene, pyrene, benz[a]anthracene, and chrysene) was available. Moreover, the ETS role in determining the total PAH burden, taking in account other potential PAH sources, was investigated using multiple regression models.

## 2. Materials and Methods

### 2.1. Study Population and Sample Collection

The data presented in this paper have been collected through a cross-sectional biomonitoring study on exposure to emissions from a local urban-waste incineration plant [19]. Recruitment, interviewing, and sampling took place between November 2012 and April 2013. The study population consisted of volunteer adult participants (18–70 years) from the general population of Modena, a medium-sized town in Northern Italy (Emilia-Romagna region, Italy). Eligible subjects were randomly selected from the population base, which comprises approximately 40% of the town population. Sampling method implied stratification by gender, age group (18–34, 35–49 and 50–69 years), and exposure. The study sample was similar to the source population (the town population) in terms of sex, age, and citizenship based on a comparison with data of the population register and of the health surveillance system. The number of subjects to be included was calculated to detect a significant difference of at least 20% in biomarker levels comparing the most exposed to the least exposed subject; this results in about 500 individuals. Sampling comprised the selection of three replacements for each subject, belonging to the same sampling stratum. Detailed information on the recruitment criteria and procedure was described previously [17,18].

Invitations to participate in the study were sent out by post. Individuals were supplied with a study pack containing the invitation letter, the questionnaire, a disposable polyethylene bottle, and the instruction to collect a spot urine sample from the first void of the day. Subjects were telephonically contacted about one week after the dispatch of the invitation letter and those answering positively were invited to the Local Health Unit to provide the biological sample and to complete the questionnaire on personal and lifestyle characteristics. Urine samples were immediately refrigerated at 4 °C and delivered to the laboratory, where they were kept at −20 °C in the dark. Not-respondents and refusals were substituted in an appropriate way to maintain the stratification homogeneity.

All participants were informed about the aims of the research and signed an informed consent form. The study was approved by the ethics committee of Modena. The study has been carried out in accordance with the Declaration of the World Medical Association (Declaration of Helsinki) for experiments involving humans (http://www.wma.net/e/policy/b3.htm).

### 2.2. Questionnaire for Assessment of Active or Environmental Exposure to Tobacco Smoke

Participants completed a detailed questionnaire about current active and passive smoke exposure [18]. The used items were adapted from available questionnaires used in large population surveys and had been verified in our previous studies [18,20]. The questionnaire was reviewed by a trained interviewer at the moment of urine sample collection. In particular, to classify current active exposure to tobacco smoke, the following questions were asked: current active tobacco smoking (yes/no), smoking product (cigarette/cigar/pipe/e-cigarette/other), product commercial name, weekly and daily smoking intensities, and smoking environment (only open places/only closed places/both open and closed places). To classify current ETS exposure, the following questions were asked: living with smokers (yes/no), cohabitants smoked in the house (yes/no), working with smokers (yes/no), coworkers smoked in the same room (yes/no), and daily ETS exposure within the last week (yes/no). If a participant answered “yes” to the last question, then information on ETS duration (how many days/week; how many hours/week; how many hours/day), smoking type (cigarette/cigar/pipe smoke), and environment (home/work/leisure time/car; open/closed places) was collected. Moreover, the time elapsed from the last smoke exposure to urine collection was ascertained at the moment of urine collection.

### 2.3. Quantification of COT-U Level

Urinary cotinine (COT-U) was quantified by a liquid chromatography coupled to mass spectrometry with triple quadrupole (LC-MS/MS) method [20]. The limit of quantification (LOQ) was 0.1 µg/L, range of linearity was 0.1–4000 µg/L, precision was <2%, and accuracy was ±1% of the theoretical values. Subjects with COT-U >30 µg/L were classified as active smokers [18,20].

### 2.4. Urinary PAHs and 1-OHPYR Analysis

Prior to analysis, urinary samples were left at room temperature until completely thawed. After shaking, two aliquots for the determination of urinary PAHs and 1-OHPYR were transferred to the analysis vials and underwent the respective analytical procedures, as described below.

Urinary PAHs [naphthalene (U-NAP), acenaphthylene (U-ACY), acenaphthene (U-ACE), fluorene (U-FLU), phenanthrene (U-PHE), anthracene (U-ANT), fluoranthene (U-FLT), pyrene (U-PYR), benz[a]anthracene (U-BaA), and chrysene (U-CHR)] were determined by solid-phase microextraction (SPME) followed by gas chromatography coupled to mass spectrometry with triple quadrupole (GC-MS/MS) [21]. Prior to analysis, samples were spiked with a mixture containing 10 deuterated analogues as internal standards. Overall, LOQs were in the 0.2–5.4 ng/L range, within-run and between-run precision, calculated on internal quality standards and expressed as the coefficient of variation (CV%), were both <20%, and accuracy varied from 91 to 120%.

1-Hydroxypyrene (1-OHPYR) was determined by LC-MS/MS (TSQ Quantum Access, Thermo Scientific, Rodano, Italy), after enzymatic hydrolysis and solid phase extraction. Analysis was performed in the presence of a deuterated internal standard. LOQ was 0.05 µg/L, while precision and accuracy, calculated on internal quality standards (0.5 and 2 µg/L), were <10% and in the 96–99% range, respectively. Accuracy, calculated on low (NIST SRM 3673 Organic contaminants in non-smokers’ urine, 0.03 µg/L) and high (NIST SRM 3672 Organic contaminants in smokers’ urine, 0.17 µg/L) standard reference materials was 79 and 107%, respectively.

### 2.5. Urinary Creatinine

Urinary creatinine was determined using Jaffe’s colorimetric method [22]. The creatinine value was used to assure sample validity, excluding samples with excessive physiologic dilution or concentration according to the 0.3 g/L ≤ creatinine ≤3.0 g/L range [23]. Moreover, creatinine was introduced in the regression models as an independent variable [24].

### 2.6. Statistical Analysis

Statistical analysis was performed by using the IBM SPSS (ver. 24.0 for Windows; SPSS Statistics, IBM Italia, Segrate, Italy) and by the STATA 11 software packages (Stata Corp LP, College Station, TX, USA). The median and percentiles of COT-U and PAH biomarkers were used to describe the non-parametric distributions in all subjects and in subgroups stratified by ETS exposure. Active smoking was defined as self-reported current smoking or COT-U levels ≥ 30 µg/L. Among correctly identified nonsmokers (individuals self-classified as nonsmokers and with COT-U < 30 µg/L), subjects were classified as ETS exposed alternatively based on the questionnaire, when daily ETS exposure within the last week was reported in the questionnaire (ETS_QUEST_), or based on a COT-U value of 1.78 (90% confidence interval 1.75–1.80) µg/L, previously derived as upper reference value in not ETS exposed Italian adults (ETS_COT_) [18].

Pearson’s correlations were used to test the associations between variables. The Student’s *t* test for independent samples was used on decimal log-transformed data for the univariate evaluation of differences between groups. The chi-square test was used to compare the percentage distributions among groups.

For each biomarker, three multivariate linear regression models were estimated to study the influence of ETS exposure on PAH body burden. ETS exposure was introduced in the regression models alternatively as a continuous variable (not transformed COT-U, µg/L, Model 1) or as a dichotomous variable (ETS_COT_ in Model 2 or ETS_QUEST_ in Model 3). Considering the large number of confounding factors that may influence PAH exposure, an extensive pool of complementary information including diet, environmental sources of exposure, occupational exposure, characteristic of the residence, atmospheric parameters, and personal habits, was collected by a detailed questionnaire, as described previously [17]. Covariates included in the finals models were those found to have a significant effect in preliminary regression analysis or known to affect biomarkers based on the pertinent literature. Some covariates were included in models as fixed variables, regardless of the statistical significance: gender, age, education level (low/high), citizenship (Italian/others), body mass index, creatinine, residence zone (rural, industrial, urban, mixed), daily temperature, precipitation, outdoor traffic exposure (low, medium, high), heating exposure (NOx), time spent at home (h/day), residential distance from major roads, and occupational exposure to PAHs (yes/no). The sampling day was introduced as a spline variable (with up to three knots) to control for exposure variability.

To account for the impact of a potential selection bias, the Inverse Probability Weighting (IPW) was calculated; the weighting allows to correct for the likelihood of adherence estimated by socio-demographic variables (gender, age, citizenship), which were the only markers available in the administrative database both for respondents and non-respondents. Unfortunately, the education level, which is known to be related to compliance, as well as the other used socio-demographic variables, was not available in the administrative database.

In the regression models, PAH biomarkers were transformed according to the distribution characteristics by Box-Cox transformation. A logarithmic transformation was applied if the lambda of the Box-Cox transformation was equal to zero. For biomarker values below the LOQ the actual values were used. Outliers, defined as samples with biomarker levels above the 99th percentile, were excluded from statistical analysis. Samples with creatinine concentrations above 3 g/L or below 0.3 g/L were excluded as well.

## 3. Results

### 3.1. Study Population

**Figure 1:**
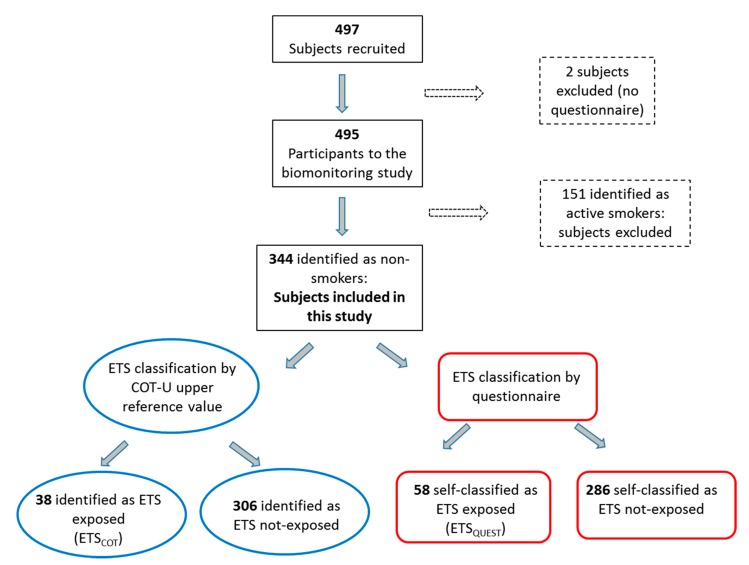
Participant flow-chart.

Figure 1 shows the participant flow-chart. Among 497 individuals recruited to the biomonitoring study (response rate 53.5%, non-availability rate 19.2%, refusal rate: 27.3%), two participants were excluded from the analysis because they did not complete the questionnaire, while 151 were identified as active smokers and were excluded as well. Finally, 344 individuals were identified as nonsmokers and were included in this study. Their mean age (minimum-maximum) was 45 (18–69) years, 183 (53%) participants were female, 324 (94%) were white individuals from Italy or other European countries (Table 1).

### 3.2. ETS Exposure

In all subjects, the median (5th–95th percentile) COT-U was 0.47 (<0.1–3.91) µg/L. According to the questionnaire, 58 (17%) subjects reported daily ETS exposure within the last week (ETS_QUEST_), whereas 286 (83%) reported no daily ETS exposure (Figure 1). Their median COT-U levels were 1.38 (<0.1–9.06) and 0.39 (<0.1–1.78) µg/L, respectively (*p* < 0.001). Subjects reporting ETS exposure were younger than those not reporting exposure (40 vs. 46 years, *p* = 0.004), were predominantly female (60% female vs. 40% male), and had a lower education level (Table 1). ETS_QUEST_ subjects reported mean ETS exposure for 2 h/day, 10 h/week, and 5 day/week.

Based on the COT-U value of 1.78 µg/L, 38 (11%) individuals were identified as ETS exposed (ETS_COT_) and 306 (89%) as not-ETS exposed (Figure 1). Their median COT-U levels were 3.63 (1.8–17.39) and 0.40 (<0.1–1.32) µg/L, respectively (*p* < 0.001). No difference in terms of age, gender, and education was found between ETS_COT_ and not-ETS exposed subjects (Table 1). In ETS_COT_ subjects, ETS duration was significantly longer than in not-ETS exposed subjects (3 vs. 1 h/day, 18 vs. 6 h/week, and 6 vs. 4 day/week, respectively).

Twenty-three subjects reported to be ETS exposed and had COT-U levels higher than 1.78 µg/L.

### 3.3. PAH Exposure and ETS

Table 2 reports the results of urinary PAH and 1-OHPYR levels in all subjects and in subjects stratified by ETS exposure based on self-classification by questionnaire and on COT-U excretion. Considering all subjects together, urinary PAHs were detected at least in 65% of samples, beside U-BaA, that was detected only in 18% of samples. U-NAP, U-PHE, and U-ANT were detected in all samples. 1-OHPYR was quantified only in 33% of subjects.

In subjects stratified by ETS exposure based on self-reporting, the percentage of analytes above the LOQ was similar between ETS_QUEST_ and not-exposed individuals. Urinary PAH or 1-OHPYR levels were not different comparing ETS_QUEST_ vs. not-exposed individuals; only for U-ANT, slightly higher levels were observed in ETS_QUEST_ than in not-exposed subjects (2.0 vs. 2.2 µg/L, *p* = 0.102).

In subjects stratified by ETS exposure based on COT-U excretion, the percentage of analytes above the LOQ was similar between ETS_COT_ and not-exposed individuals; only for U-ACE and 1-OHPYR a slightly higher percentage of samples above the LOQ was found in ETS_COT_ than in not-exposed subjects (89 vs. 78%, *p* = 0.102; and 42 vs. 32% *p* = 0.213, respectively). Significantly higher levels of 1-OHPYR (*p* = 0.037), U-FLU (*p* = 0.028), U-ANT (*p* = 0.028), and U-PYR (*p* = 0.052) were found in ETS_COT_ than in not-exposed individuals.

### 3.4. Contribution of ETS to PAH Exposure

In all subjects, COT-U levels were significantly, or marginally significantly, correlated with 1-OHPYR (Pearson’s *r* = 0.121, *p* = 0.024), U-ANT (*r* = 0.128, *p* = 0.017), U-FLT (*r* = 0.094, *p* = 0.083), U-PYR (*r* = 0.106, *p* = 0.051), and U-CHR (*r* = 0.114, *p* = 0.035).

Table 3 shows the results of the adjusted linear regression models in detail. Only beta-coefficients (β) of ETS exposure, together with the 95% confidence intervals, are shown regardless of the significance level. Due to the outlier exclusion, the sample size was different for each PAH biomarker and ranged from 287 (1-OHPYR) to 313 (U-ACY, U-FLU, and U-FLT). The complete regression model, showing beta-coefficients for all the independent variables, is shown in Supplementary Table S1 for Model 1. Multivariate regression models were performed for each analyte, except for U-BaA, given the high proportion of samples with levels below the LOQ for this biomarker.

When ETS exposure was introduced in the regression model as a continuous variable (unadjusted COT-U levels, Model 1), ETS was associated to 1-OHPYR (beta 0.042, *p* = 0.026), with a 4% percentage increment for each 10-fold increase in cotinine excretion. The other variables associated to 1-OHPYR were creatinine (beta = 0.680, *p* < 0.001), coffee intake (beta= 0.267, *p* = 0.074), education level (beta = 0.243, *p* = 0.069), and the sampling day (beta = 0.007, *p* < 0.001). A marginal association between ETS and U-ANT was also suggested by the model (beta 0.015, *p* = 0.134). Other variables positively affecting PAH biomarkers in this model were the sampling day (1-OHPYR, U-ACY, U-ANT, and U-CHR), the exposure to the waste incineration emissions (U-FLU), gender (U-PHE and U-CHR), the education level (1-OHPYR), the citizenship (U-NAP, U-FLU, U-PHE, U-FLT, U-PYR, and U-CHR), creatinine (1-OHPYR, U-PHE, and U-ANT), living in an industrial or urban area (U-NAP), daily precipitation (U-ACY), medium or high traffic exposure (U-ACY, U-FLU, and U-ANT), heating emission exposure (U-ACY and U-FLU), the presence of mold on the residence wall (U-NAP, U-ACY, U-FLU, U-PHE, U-ANT, U-FLT), the use of chemicals in domestic environments (U-ANT), the use of medication (U-ACE), or nutritional supplement ((U-FLT), and some specific food such as tuna (U-ACE), coffee (1-OHPYR), and wine (U-NAP) (Supplementary Table S1).

When ETS exposure was introduced in the regression model as a dichotomous variable based on COT-U excretion (ETS_COT_, Model 2), only U-FLU resulted influenced by ETS (beta = 0.126, *p* = 0.061). The other variables associated to U-FLU were the citizenship (beta = 0.126, *p* = 0.067, the sampling day (beta = 0.096, *p* < 0.001), the exposure to the waste incineration emissions (level 2, beta = 0.147, *p* = 0.027, and level 3 = 0.166, *p* = 0.008), age (beta = 0.003, *p* = 0.092), creatinine (beta = −0.064, *p* = 0.071), living in a rural area (urban area, beta = −0.230, *p* = 0.004, and mixed area, beta = −0.138, *p* = 0.065), medium traffic exposure (beta = 0.126, *p* = 0.006), heating emission exposure (beta = 0.009, *p* = 0.002), and the presence of mold on the residence wall (beta = 0.117, *p* = 0.028).

When ETS exposure was introduced in the regression model as a dichotomous variable based on self-classification by questionnaire (ETS_QUEST_, Model 3), none of the PAH biomarker resulted associated to ETS.

For both Model 2 and Model 3, the results regarding other variables affecting PAH biomarkers were very similar to what is shown for Model 1 in Supplementary Table S1.

## 4. Discussion

### 4.1. Biomarkers of ETS and PAH Exposure

In this study, urinary cotinine was measured in samples from 344 individuals from a representative group of the general population. Among the investigated subjects, 58 (17%) reported being daily ETS exposed (ETS_QUEST_) (Table 1). Median COT-U levels in these subjects were higher than those in subjects not reporting ETS exposure (1.38 vs. 0.39 µg/L, *p* < 0.001) and consistent with those found in studies conducted in countries where a smoking-ban in public places has been implemented [25,26]. As several variables may affect ETS exposure (i.e., source, duration, intensity) and certain individuals may be particularly prone to report it, the use of the upper reference value of CUT-U (1.78 µg/L) as a cut-off value allowed us to identify those individuals who were exposed to high ETS level (ETS_COT_). Median COT-U levels (3.63 µg/L) in these subjects were similar to those previously reported in non-smoking adults before the smoking-ban was implemented [12,27,28,29].

The exposure to PAHs was evaluated by measuring urinary 1-OHPYR and ten unmetabolized PAHs (Table 2). In all subjects, 1-OHPYR was quantifiable only in 33% of the samples, with median levels <0.05 µg/L and well below the reference value for the Italian population (<0.3 μg/L in non-smokers and <0.7 μg/L in smokers) [30]. Urinary PAHs, except U-BaA, were quantifiable in the large majority of samples, even though median levels of all analytes were very low (ng/L order of magnitude). Not so many studies reported the measurement of urinary PAHs in the general population: the levels here found were similar or lower than those reported for adults in the town of Modena [31] and in a small group of adolescents participating in the Flemish Environment and Health Study [32], and much lower than those of non-smoking adults living in an industrial polluted area in Poland [33]. Altogether, PAH biomarkers measured in this study are indicative of very low PAH exposure.

### 4.2. ETS and PAH Exposure

The possible role of ETS in determining PAH intake has been seldom investigated. Higher levels of PAH metabolites in ETS exposed adults than in not-exposed were reported for 1-OHPYR with a linear relationship between 1-OHPYR excretion and duration of ETS exposure [34], for hydroxylated metabolites of phenanthrene and again 1-OHPYR in highly ETS exposed subjects [12], and for hydroxylated metabolites of phenanthrene, fluorene, and pyrene in the U.S. general population participating in the 1999–2002 National Health and Nutrition Examination Survey [11]. More recently, 1-OHPYR was found higher in adults exposed to ETS during the weekend or more than 4 h/day along the week than in not-exposed adults participating in a human biomonitoring program on a national scale in Spain [35]. Among several PAH biomarkers investigated in this study, the levels of 1-OHPYR, U-FLU, U-ANT, and U-PYR were significantly higher in subjects stratified as ETS-exposed based on COT-U excretion (ETS_COT_ subjects) (Table 2). Thus, consistently with previous reports, higher exposure to PAHs was evident only in highly ETS-exposed subjects.

As regards the exposure to specific PAHs, a very interesting result of this study is that U-FLU was higher in ETS_COT_ subjects than in not-ETS exposed subjects (Table 2). Fluorene, which is a low-molecular weight PAH with three aromatic rings like anthracene, resulted among the compounds more contributing to mainstream smoke composition (as ng/cigarette) and among the most abundant in the gas phase of tobacco smoke [4]. Previous studies indicated fluorene hydroxylated metabolites as more specific and selective biomarkers than 1-OHPYR to discriminate PAH exposure in smokers and nonsmokers [10,36]. Our present results suggest that U-FLU may be indicative of exposure to low-molecular weight compounds in ETS-exposed subjects too.

Positive correlations were found between COT-U and some of the measured PAH biomarkers, including 1-OHPYR, U-ANT, U-FLT, U-PYR, and U-CHR. It is interesting that 4-ring PAHs, such as fluoranthene, pyrene and chrysene, are among the most important contributors to side-stream smoke [3]. This result is consistent with a previous study where a correlation between environmental chrysene exposure and urinary cotinine was shown [12] and it highlights the existence of a relation between ETS exposure and particle-bound PAHs.

### 4.3. Role of ETS in Determining PAH Exposure

Given that PAHs are originated from multiple sources and co-exposure is very common, especially in not-occupationally exposed individuals, the regression analysis enabled us to evaluate the role of ETS exposure in determining PAH exposure (Table 3 and Supplementary Table S1). The regression models suggested a weak contribution of ETS to PAH intake and only when ETS was defined by COT-U excretion (both as continuous variable in Model 1, and as a dichotomous variable in Model 2). This underlines that the questionnaire may be not an adequate tool to define ETS exposure for the wide variability of ETS exposure occurrence and the consequent individual’s difficulty in recognizing and/or recalling it [18].

Among the measured biomarkers, significant or marginally significant associations with COT-U were found only for 1-OHPYR (with a percentage increment of 4% for each 10-fold increase in cotinine excretion) and for U-FLU, partly confirming the results of the univariate analysis.

Moreover, the regression analysis highlighted that ETS is a minor source of PAH exposure among other sources: other contributors to 1-OHPYR excretion were creatinine, the education level, and the intake of coffee, while other contributors to U-FLU excretion were the exposure to the waste incinerator emissions, the residence area, the exposure to traffic, and the presence of mold on the residence wall (Supplementary Table S1). While some associations may be expected, such as those found with diet, traffic exposure, and the residence area, others, among which the presence of mold on the residence wall, the education level, and the citizenship may be not so obvious and may be proxies of other factors (i.e., different diet, cooking habits, lifestyle, and residence characteristics).

### 4.4. Limitations and Strengths

Some limitations may be identified in this study, such as the lack of PAH personal air measurements that would have allowed making associations between PAH exposure from ETS and biomarkers. Moreover, even if the original sample population consisted of about 500 individuals, with a sample size adequate to observe a significant difference in the biomarkers levels, the number of ETS exposed subjects was actually small, which may have influenced the robustness of the present findings. The strength of this study is that several PAH biomarkers were investigated simultaneously on a representative group of the general population, giving the opportunity to better characterize the exposure profile. Few studies reported the association between ETS and PAH exposure, and only hydroxylated metabolites were investigated [11,12], whereas this is the first time that unmetabolized PAHs have been studied to this aim. Moreover, other potential sources of possible PAH exposure were carefully accounted for by regression models allowing us to investigate the role of ETS in determining PAH body burden.

## 5. Conclusions

In conclusion, this study highlighted a weak, but significant, contribution of ETS exposure to the PAH body burden, as shown by 1-OHPYR and fluorene increases in ETS exposed individuals. On the other hand, our results suggest that ETS exposure experienced by subjects in this study represents a minor source of exposure to PAHs.

## Figures and Tables

**Table 1:** Characteristics of participants (*n* = 344), stratified by environmental tobacco smoke (ETS) exposure based on COT-U excretion (ETS_COT_: COT-U > 1.78 µg/L = ETS exposed) and on self-classification by questionnaire (ETS_QUEST_).

Characteristics		All Subjects *N* (%)	ETS Exposure by Questionnaire *N* (%)			ETS Exposure by COT-U *N* (%)		
			No-ETS Exposure	ETS Exposure (ETS_QUEST_)	*p* *	No-ETS Exposure	ETS Exposure (ETS_COT_)	*p* *
		344	286 (83)	58 (17)	-	306 (89)	38 (11)	-
Age (years) Mean (minimum-maximum)		45 (18–69)	46 (18–69)	40 (18–68)	0.004	45 (18–69)	42 (18–68)	0.122
Age (class) *N* (%)	18–34	96 (28)	70 (2)	26 (45)	0.004 ^χ^	81 (26)	15 (39)	0.240 ^χ^
	35–49	111 (32)	99 (35)	12 (21)		101 (33)	10 (26)	
	50–69	137 (40)	117 (41)	20 (34)		124 (41)	13 (34)	
Gender *N* (%)	Male	161 (47)	138 (48)	23 (40)	0.191 ^χ^	163 (53)	20 (53)	0.941 ^χ^
	Female	183 (53)	148 (52)	35 (60)		143 (47)	18 (47)	
Education *N* (%)	Primary school	13 (9)	9 (3)	4 (7)	0.077 ^χ^	10 (3)	3 (8)	0.361 ^χ^
	Secondary school	82 (24)	63 (22)	19 (33)		71 (23)	11 (29)	
	High school	144 (42)	122 (43)	22 (38)		131 (43)	13 (34)	
	Graduated	103 (30)	90 (31)	13 (22)		93 (30)	10 (26)	
Income (€/month) *N* (%)	No income	3 (1)	3 (1)	0 (0)	0.452 ^χ^	3 (1)	0	0.700 ^χ^
	<1000	24 (7)	20 (7)	4 (7)		22 (7)	2 (5)	
	1001–2000	94 (27)	75 (26)	19 (33)		80 (26)	14 (37)	
	2001–3000	99 (29)	78 (27)	21 (36)		91 (30)	8 (21)	
	3001–5000	76 (22)	68 (24)	8 (14)		68 (22)	8 (21)	
	>5000	23 (7)	21 (7)	2 (3)		21 (7)	2 (5)	
	Don’t know/missing	25 (7)	18 (6)	4 (7)		21 (7)	4 (10	
Ethnicity	European whites	324 (94)	271 (95)	53 (91)	0.311 ^χ^	289 (94)	35 (92)	0.416 ^χ^
	Non-European whites	8 (2)	7 (2)	1 (2)		7 (2)	1 (3)	
	African blacks	5 (1)	4 (1)	1 (2)		5 (2)	0 (0)	
	Others	7 (2)	4 1)	3 (5)		5 (2)	2 (5)	
ETS duration (h/day) Mean (minimum-maximum)		-	-	2 (0–10)	-	1 (0–4)	3 (0–10)	0.054
ETS duration (h/week) Mean (minimum-maximum)		-	-	10 (0–70)	-	6 (0–28)	18 (0–70)	0.035
ETS duration (day/week) Mean (minimum-maximum)		-	-	5 (1–7)	-	4 (1–7)	6 (2–7)	0.002
COT-U (µg/L) Median (5th–95th percentile)		0.47 (<0.1–3.91)	0.39 (<0.1–1.78)	1.38 (<0.1–9.06)	<0.001	0.40 (<0.1–1.32)	3.63 (1.8–17.39)	<0.001

* = Determined by Student’s *t* or chi-square test comparing ETS-exposed vs. non-ETS exposed subjects; ^χ^ = Determined by chi-square test.

**Table 2:** Urinary 1-OHPYR and PAH levels in subjects stratified by ETS exposure based on COT-U excretion (ETS_COT_: COT-U > 1.78 µg/L) and on self-classification by questionnaire (ETS_QUEST_).

Analyte	All Subjects (*N* = 344)	ETS Exposure by Questionnaire			ETS Exposure by COT-U		
		No-ETS Exposure (*N* = 286)	ETS Exposure (ETS_QUEST_) (*N* = 58)	p *	No-ETS Exposure (*N* = 306)	ETS Exposure (ETS_COT_) (*N* = 38)	p *
	Median (5–95 percentile); % > LOQ	Median (5–95 percentile); % > LOQ	Median (5–95 percentile); % > LOQ		Median (5–95 percentile); % > LOQ	Median (5–95 percentile); % > LOQ	
1-OHPYR µg/L	<0.05 (<0.05–0.13); 33	<0.05 (<0.05–0.13); 34	<0.05 (<0.05–0.11); 31	0.395	<0.05 (<0.05–0.12); 32	<0.05 (<0.05–0.17); 42	0.037
U-NAP ng/L	24.7 (13.4–78.7); 100	24.6 (13.0–80.0); 100	25.1 (14.2–65.3); 100	0.742	24.7 (13.4–80.0); 100	25.1 (12.5–63.3); 100	0.516
U-ACY ng/L	0.5 (<0.3–1.5); 95	0.5 (<0.3–1.5); 95	0.6 (<0.3–1.7); 95	0.517	0.5 (0.3–1.5); 95	0.6 (<0.3–1.5); 92	0.937
U-ACE ng/L	0.9 (<0.6–3.4); 79	0.9 (<0.6–3.4); 78	1.0 (<0.6–3.0); 85	0.889	0.9 (<0.6–3.5); 78	0.9 (<0.6–2.1); 89	0.768
U-FLU ng/L	1.5 (0.9–2.9); 96	1.5 (0.9–2.8); 96	1.5 (1.0–2.9); 97	0.798	1.5 (0.9–2.8); 96	1.7 (1.0–3.2); 100	0.028
U-PHE ng/L	7.2 (4.7–14.6); 100	7.2 (4.6–14.1); 100	7.1 (5.0–17.2); 100	0.566	7.1 (4.7–14.9); 100	7.7 (5.0–13.5); 100	0.274
U-ANT ng/L	2.1 (0.9–3.1); 100	2.0 (0.9–3.1); 100	2.2 (0.8–5.7); 100	0.102	2.1 (0.9–3.1); 100	2.3 (1.0–3.3); 100	0.028
U-FLT ng/L	0.7 (<0.6–1.3); 72	0.7 (<0.6–1.3); 72	0.6 (<0.6–1.4); 69	0.728	0.7 (<0.6–1.2); 71	0.8 (<0.6–1.5); 73	0.340
U-PYR ng/L	0.6 (<0.4–1.1); 92	0.6 (<0.4–1.1); 91	0.6 (<0.4–1.3); 95	0.323	0.6 (<0.4–1.1); 92	0.7 (<0.4–1.3); 89	0.052
U-BaA ng/L	<0.3 (<0.3–0.8); 18	<0.3 (<0.3–0.8); 19	<0.3 (<0.3–1.4); 17	0.809	<0.3 (<0.3–0.8); 19	<0.3 (<0.3–2.1); 13	0.716
U-CHR ng/L	0.2 (<0.2–0.7); 65	0.2 (<0.2–0.6); 64	0.2 (<0.2–1.7); 67	0.223	0.2 (<0.2–0.7); 65	0.2 (<0.2–1.4); 66	0.398

* = Determined by Student’s *t* test comparing ETS-exposed vs. non-ETS exposed subjects.

**Table 3:** Results of multiple linear regression analyses for predicting the levels of urinary PAHs and 1-OHPYR in study subjects. ETS exposure was introduced in the regression models alternatively as a continuous variable (unadjusted COT-U levels, Model 1), or as a dichotomous variable (ETS_COT_ in Model 2, and ETS_QUEST_ in Model 3). Beta-coefficients (β) of ETS exposure, together with the 95% confidence intervals, are shown regardless of the significance level. The complete regression model is shown in Supplementary Table S1 for Model 1.

Analyte	Statistics ^a,b^	Model 1	Model 2	Model 3
		COT-U (µg/L)	ETS_COT_	ETS_QUEST_
1-OHPYR *	Beta (std)	0.043 (0.019)	0.078 (0.194)	−0.017 (0.144)
	95%CI	0.005–0.080	−0.304–0.461	−0.300–0.266
	*p*	0.026	0.687	0.906
U-NAP	Beta (std)	0.002 (0.003)	0.014 (0.025)	0.005 (0.024)
	95%CI	−0.004–0.009	−0.035–0.064	−0.043–0.053
	*p*	0.476	0.572	0.842
U-ACY	Beta (std)	−0.003 (0.012)	0.076 (0.100)	−0.049 (0.087)
	95%CI	−0.027–0.020	−0.119–0.271	−0.221–0.123
	*p*	0.781	0.445	0.573
U-ACE *	Beta (std)	−0.003 (0.013)	0.003 (0.103)	0.060 (0.106)
	95%CI	−0.028–0.023	−0.200–0.205	−0.149–0.270
	*p*	0.821	0.979	0.572
U-FLU	Beta (std)	0.003 (0.011)	0.126 (0.067)	0.035 (0.054)
	95%CI	−0.017–0.024	−0.006–0.258	−0.071–0.140
	*p*	0.743	0.061	0.520
U-PHE	Beta (std)	−0.000 (0.001)	0.000 (0.010)	−0.008 (0.011)
	95%CI	−0.003–0.002	−0.0189–0.019	−0.030–0.015
	*p*	0.737	0.980	0.503
U-ANT	Beta (std)	0.015 (0.010)	0.024 (0.087)	0–0.018 (0.073)
	95%CI	−0.005–0.034	−0.147–0.194	−0.162–0.126
	*p*	0.134	0.784	0.804
U-FLT	Beta (std)	0.009 (0.008)	0.0480 (0.067)	−0.032 (0.053)
	95%CI	−0.008–0.0247	−0.084–0.179	−0.135–0.071
	*p*	0.299	0.474	0.545
U-PYR	Beta (std)	0.004 (0.007)	0.040 (0.063)	0.000 (0.049)
	95%CI	−0.010–0.017	−0.085–0.163	−0.097–0.097
	*p*	0.616	0.534	0.999
U-CHR *	Beta (std)	−0.015 (0.013)	−0.007 (0.100)	−0.056 (0.092)
	95%CI	−0.041–0.011	−0.204–0.190	−0.238–0.126
	*p*	0.254	0.944	0.547

^a^ = In model 2 and 3, not-exposed subjects were the reference group. ^b^ = *p* values represent significance of ETS exposure for each PAH biomarker. * = logarithmic transformation.

## Supplementary Materials

The following are available online at http://www.mdpi.com/1660-4601/15/6/1156/s1, Table S1: Estimates of regression models using urinary PAHs as dependent variables. Beta-coefficients (β) of COT-U are shown regardless of the significance level, whereas only β values below the 0.10 significance level are illustrated for the other covariates.
