# Supplementary material for: ETS Exposure and PAH Body Burden in Nonsmoking Italian Adults
Source: Int J Environ Res Public Health. 2018 Jun 1;15(6):1156. doi: 10.3390/ijerph15061156 (PMC6025440; doi:10.3390/ijerph15061156)
Supplement: Supplementary file 1 [file ijerph-15-01156-s001.zip › ijerph-292995-SI.pdf]

Supplementary Table 1. Estimates of regression models using urinary PAHs as dependent variables. Beta-coefficients (β) of COT-U are shown regardless of the significance level, whereas only β values below the 0.10 significance level are illustrated for the other covariates.

	PAH	1-OHPYR	Nap	Acy	Ace	Flu	Phe	Ant	Flt	Pyr	Chr
	**Sample size**	287	294	313	305	313	301	306	313	311	310
	**Box & Cox λ transformation**[a]	0.06	-0.34	-0.28	-0.08	0.27	-0.75	0.71	0.11	0.14	-0.09
	**R^2**	0.36**	0.33**	0.14**	0.14*	0.23**	0.22**	0.48**	0.11*	0.08	0.23**
		**β**	**β**	**β**	**β**	**β**	**β**	**β**	**β**	**β**	**β**
	**Sampling day**	0.007**		0.002**	-0.002**			0.009**	ns	ns	0.005**
	Day 0		-0.056**			ns	0.008*	ns			
	Day 1		ns			0.095**	0.029**	ns			
	Day 2		-0.040**			ns	ns	ns			
	**SWI exposure (ref. level 1)**										
	level 2	ns	-0.061*	-0.179*	ns	ns	ns	ns	ns	ns	-0.295**
Fixed variates	level 3	ns	ns	ns	ns	0.152*	ns	ns	ns	ns	-0.255**
	level 4 (highest)	ns	ns	ns	ns	0.175**	ns	ns	ns	ns	ns
	**Gender (ref. male)**	ns	ns	ns	ns	ns	0.018*	ns	ns	ns	0.219**
	**Age, years**	-0.011**	ns	ns	ns	ns	ns	ns	ns	ns	ns
	**Education level (ref. low)**	0.243*	ns	ns	ns	ns	ns	ns	ns	ns	ns
	**Citizenship (ref. Italian)**	ns	0.109**	ns	ns	0.126*	0.053**	ns	0.189**	0.150**	0.284*
	**BMI, kg/cm2**	ns	ns	ns	ns	ns	ns	ns	ns	ns	ns
	**Cotinine, µg/L**	0.043**	0.002	-0.003	-0.003	0.003	-0.000	0.015	0.009	0.004	-0.015
	**Creatinine, g/L**	0.680**	-0.049**	ns	ns	-0.059**	0.028**	0.093*	ns	ns	ns
	**Residence zone (ref. rural)**										
	industrial	ns	0.205**	0.378**	ns	ns	ns	ns	ns	ns	ns
	urban	ns	0.076*	ns	ns	-0.226**	ns	ns	ns	ns	ns

	mixed	ns	0.072*	ns	ns	-0.130*	ns	ns	ns	ns	ns
	**Temperature, °C**	ns	ns	ns	-0.031**	ns	-0.003**	ns	0.010*	ns	ns
	**Precipitation, mm**	ns	ns	0.026*	ns	ns	ns	-0.021*	ns	ns	ns
	**Outdoor traffic exposure (ref. low)**										
	medium	ns	ns	ns	ns	0.127**	ns	0.109*	ns	ns	ns
	high	ns	ns	0.227*	ns	ns	ns	ns	ns	ns	ns
	**Heating exposure as NOx µg/m3**	ns	ns	0.010*	ns	0.009**	ns	ns	ns	ns	ns
	**Time spent at home, h**	ns	ns	ns	ns	ns	ns	ns	ns	ns	ns
	**Residential distance from major road, m**	ns	ns	ns	ns	ns	ns	ns	ns	ns	ns
	**Occupational exposure to PAH**	ns	ns	ns	ns	ns		ns	ns	ns	ns
**PAH-specific variates**	**Mold on the residence wall**	ns	0.049*	0.168*	ns	0.117*	0.018*	0.199**	0.109*	ns	ns
	**Medication**[b]			ns	0.179*						
	**Nutritional supplement**[b]						ns		0.098*	ns	ns
	**Char-grilled food**[b]	ns		ns	ns			-0.114*			
	**Tuna**[b]			ns	0.360**						
	**Paint use**[b]						ns	0.304*			ns
	**Coffee**[b]	0.267*						ns		ns	ns
	**wine**[b]		0.042**								
	**Whole cereals**[b]									ns	ns
	**fish**[b]										ns

ns = not significant

** p-value <0.05

*p-value <0.1

[a] Logarithmic transformation if λ of the Box-Cox transformation equals to zero.

[b] Previous week
